# Inflammatory markers and exposure to airborne particles among workers in a Swedish pulp and paper mill

**DOI:** 10.1007/s00420-016-1119-5

**Published:** 2016-02-13

**Authors:** Håkan Westberg, Karine Elihn, Eva Andersson, Bodil Persson, Lennart Andersson, Ing-Liss Bryngelsson, Cathe Karlsson, Bengt Sjögren

**Affiliations:** Department of Occupational and Environmental Medicine, Örebro University Hospital, 701 85 Örebro, Sweden; Department of Applied Environmental Science, Atmospheric Science Unit, Stockholm University, 106 91 Stockholm, Sweden; Department of Occupational and Environmental Medicine, Institute of Laboratory Medicine, University Hospital, 221 85 Lund, Sweden; Occupational and Environmental Medicine, Sahlgrenska University Hospital, Box 414, 405 30 Göteborg, Sweden; Billerud AB, 664 28 Grums, Sweden; Department of Occupational and Environmental Medicine, Linköping University Hospital, 581 85 Linköping, Sweden; Work Environment Toxicology, Institute of Environmental Medicine, Karolinska Institutet, 171 77 Stockholm, Sweden

**Keywords:** C-reactive protein (CRP), Fibrinogen, Interleukins (IL-1b, IL-6, IL-8 and IL-10), PM_10_, PM_2.5_, Respirable dust, Serum amyloid A (SAA)

## Abstract

**Purpose:**

To study the relationship between exposure to airborne particles in a pulp and paper mill and markers of inflammation and coagulation in blood.

**Methods:**

Personal sampling of inhalable dust was performed for 72 subjects working in a Swedish pulp and paper mill. Stationary measurements were used to study concentrations of total dust, respirable dust, PM_10_ and PM_2.5_, the particle surface area and the particle number concentrations. Markers of inflammation, interleukins (IL-1b, IL-6, IL-8, and IL-10), C-reactive protein (CRP), serum amyloid A (SAA), and fibrinogen and markers of coagulation factor VIII, von Willebrand, plasminogen activator inhibitor, and D-dimer were measured in plasma or serum. Sampling was performed on the last day of the work free period of 5 days, before and after the shift the first day of work and after the shifts the second and third day. In a mixed model analysis, the relationship between particulate exposures and inflammatory markers was determined. Sex, age, smoking, and BMI were included as covariates.

**Results:**

The average 8-h time-weighted average (TWA) air concentration levels of inhalable dust were 0.30 mg/m^3^, range 0.005–3.3 mg/m^3^. The proxies for average 8-h TWAs of respirable dust were 0.045 mg/m^3^. Significant and consistent positive relations were found between several exposure metrics (PM 10, total and inhalable dust) and CRP, SAA and fibrinogen taken post-shift, suggesting a dose–effect relationship.

**Conclusion:**

This study supports a relationship between occupational particle exposure and established inflammatory markers, which may indicate an increased risk of cardiovascular disease.

## Introduction

Production of pulp and paper is an important sector of Swedish industry with around 42,000 employed. The historical production was dominated by the sulfite process, and today the sulfate process has taken the lead (Nordström et al. 1988; Persson et al. 2007). The occupational chemical exposures in this industry comprised paper and wood dust, chlorine, chlorine dioxide, formaldehyde, terpenes, cutting and lubricating oils, asbestos, and calcium carbonate (Andersson et al. 2010; Kauppinen et al. 2002; Korhonen et al. 2004). The exposures vary a lot between processes and departments (Torén et al. 1996). Further exposures include noise, microorganisms, heat, and shift work (Korhonen et al. 2004; Langseth and Kjaerheim 2006). Some studies have observed an increased risk of cardiovascular diseases among pulp and paper mill workers (Andersson et al. 2007; Hammar et al. 1992; Jäppinen and Tola 1990; Langseth and Kjaerheim 2006; Milham and Demers 1984; Persson et al. 2007; Torén et al. 1996) and others have not (Henneberger et al. 1989; Matanoski et al. 1998; Robinson et al. 1986; Sala-Serra et al. 1996; Solet et al. 1989; Wingren et al. 1991; Wong et al. 1996).

In 2010, a writing group of the American Heart Association stated that the overall evidence is consistent with a causal relationship between PM_2.5_ exposure and cardiovascular morbidity and mortality (Brook et al. 2010). These effects have been found in short-term studies, which relate day-to-day variations in air pollution and health, in long-term studies, which have followed cohorts of exposed individuals over time, and in intervention studies. There is also strong mechanistic evidence from animal and human studies supporting a systemic inflammatory response as the pathway between airborne particle exposure and cardiovascular disease. Other possible biological pathways are disturbances in the autonomic nervous system and direct effects of particles reaching the systemic circulation (Brook et al. 2010).

*The inflammatory* causal link between inhalation of particulate matter and the incidence of cardiovascular disease was launched as a hypothesis in the mid-1990 (Seaton et al. 1995; Sjögren 1997). Several inflammatory markers, such as interleukin-6, C-reactive protein (CRP), and fibrinogen, are established risk factors for cardiovascular disease (Danesh et al. 1998, 2000, 2005, 2008). Serum amyloid A (SAA) has also been proposed as a risk factor for cardiovascular disease in a battery of several biomarkers (Arant et al. 2009). Studies on qualitative and quantitative associations between particle exposure and inflammatory markers in industrial environments are sparse and have not been performed in the pulp and paper industry.

The purpose was to study the possible exposure–effect relationship between airborne particles and markers of inflammation and coagulation in workers in a pulp and paper mill.

## Methods

### Study object and group

The study was performed at a pulp and paper mill in the middle of Sweden with 850 employees. The mill is one of the largest producers of paper for packaging in Europe. The raw material is softwood as well as hardwood, and the production is based on unbleached and bleached sulfate pulp as well as neutral sulfite semi-chemical (NSSC) pulp. The products has included Kraft paper, sacks, spinning paper, white top liner (WTP), coated paper, sterile paper, sandwich and envelope paper. The departments represented were raw material supply, pulp and paper production, mixing and steam and power production. The exposures varies between different departments, but the main chemical exposures are wood and paper dust, terpenes and bleachery chlorine-based chemicals, calcium hydroxide, sulfur dioxide, hydrogen sulfide, and various reduced sulfur compounds.

The production is performed continuously during day and night in a 5-shift schedule. The shiftwork comprised 8-h for all workers. Production workers in each department, from at least two representative shifts out of a total of 5, were invited to participate in the study.

The acceptance rate varied between 50 and 90 % in different shifts. In total, 72 subjects, 62 men and 10 women, were investigated. Five workers were excluded due to infectious symptoms, and consequently 67 workers were included in the analysis. Their median age was 46 years (range 25–60 years), and the median duration of employment was 10 years (range 0–38). The shiftwork comprised 8-h for all workers. 16 subjects were current smokers, 16 ex-smokers and 35 were never smokers. The median BMI was 26.7. No personal protective masks were used, only gloves and glasses.

### Study design

The study was performed between May 2007 and February 2009. The investigation comprised aerosol as well as blood sampling the first days after a work free period of generally 5 days. Blood sampling was started in the afternoon (3–4.30 p.m.) the last day of the work free period (day 0), and continued before (4.45–5.45 a.m.) and after the shift the first day of work (day 1) and later after the shifts the second (day 2) and third day (day 3). These post-shift samples were taken between 1.30 and 2.30 p.m. This post-shift sampling study design aimed to control for circadian variation. A questionnaire was completed by all participants, containing items about current and previous working conditions, smoking habits, medication, and symptoms of respiratory irritation, infection or other inflammation, which could influence the levels of the biomarkers. Aerosol sampling was performed on the first day (day 1) for all participants.

The study was approved by the Regional Ethical Review Board, Uppsala, id number 2007/51.

### Aerosol measurements

The effects of inhalation of airborne particles have traditionally been focused on coarse and fine particles (Brunekreef and Forsberg 2005). In addition particle surface area and particle number have been discussed as agents with inflammatory impact in the lung. Consequently, several exposure metrics, comprising various particle size fractions, have been included. For practical reasons, personal exposure measurements could only be performed for inhalable dust. Other aerosol metrics were determined stationary.

The inhalable dust exposure of the participating subjects was determined with personal sampling measurement, i.e., using personal pumps carried by each worker, the first day after a work free period and the same day as the medical investigations. Personal sampling of inhalable dust was carried out as 8-h full shift samples according to standards for measurement of inhalable dust using GSP-samplers operating at 3.5 l/min, (HSE 2000).

Measuring rigs were used for stationary measurements (area measurements). These measurements were taken at different areas where the workers were present during the work shift, i.e., in the control room as well as along the surveillance tours performed at different departments in the plant. These measurements included sampling of inhalable dust using the same techniques as for the personal samples. Total dust was determined as 8-h full shift samples according to a modified gravimetric method (NIOSH 1994) using an open faced 25-mm filter cassettes (OFC), with an air flow of 2.0 l/min. Respirable dust was determined using SKC aluminum cyclones for 25-mm filters, operating at an air flow of 2.5 l/min (HSE 2000). The PM_10_ and PM_2.5_ air concentrations were determined using a Chempass system operating at 1.8 l/min (EN 1999, 2005). Particle surface area concentration was determined using an A-trak 9000 (Aero Trak Nanoparticle Aerosol Monitor) where the particle surface area is determined real-time, ranging in particle size from 10 to 1000 nm. The particle number concentrations in the size range 20–1000 nm was determined using a real-time aerosol monitoring instrument, (P-Trak 8525, Ultrafine Particle Counter).

In addition to the determined personally sampled exposures of inhalable dust presented as 8-h time-weighted averages (8-h TWAs), the corresponding exposure measures were determined for other aerosol and particle fractions based on the measurements performed in the rig. The TWA exposure regarding respirable and total dust, PM_10_, PM_2.5_, particle surface area as well as the particle number concentrations was calculated for each participant. These exposure metrics were estimated by recorded time in different departmental work areas for each worker and the measured aerosol air concentrations at stationary sampling sites representing these work areas. The time spent in different areas was recorded by individual logging of daily work schemes. The individual 8-h TWAs exposures were calculated in the following way for each participant:

$$C\left( {\text{TWA}} \right) = \left( {C_{1} \times T_{1} + C_{2} \times T_{2} + \cdots + C_{n} \times T_{n} } \right) /\left( {T_{1} + T_{2} + \cdots + T_{n} } \right)$$

*C*_1_: Air concentration during time period 1 (work area 1), stationary measurement 1
*C*_*n*_: Air concentration during time period *n* (work area *n*), stationary measurement *n*
*T*_1_: Time spent in period 1
*T*_*n*_: Time spent in period *n*

### Markers of inflammation and coagulation

The concentrations of several markers of inflammation and coagulation were determined in serum and plasma; interleukins (IL-1b, IL-6, IL-8, and IL-10), γ-interferon (IFNγ), tumor necrosis factor-α (TNFα), CRP, SAA, fibrinogen, factor VIII, von Willebrand factor (vWF), plasminogen activator inhibitor (PAI-1), and D-dimer. All blood samples were centrifuged and frozen to −70 °C. The analyses were performed at the Clinical Research Center, Örebro University Hospital and at the Department of Occupational and Environmental Medicine, Sahlgrenska University Hospital, Göteborg.

The concentrations of interleukins, IFNγ, and TNFα were analyzed with a Milliplex Human Cytokine/Chemokine Immunoassay kit (Millipore Corporation, 290 Concord Road Billerica, MA 01821, USA), the measurements were taken with Luminex 200TM recorder (12212 Technology Blvd, Austin, Texas, USA), and the results were calculated with Luminex xPONENT software.

### Statistical analysis

For descriptive purposes, the aerosol concentrations of different exposure metrics, i.e., inhalable, total, and respirable dust, PM_10_ and PM_2.5_, the particle surface area (A-Trak), and the particle number concentration (P-trak) were presented as 8-h TWAs for the full workday. Standard parameters such as arithmetic mean (AM), standard deviation (SD), geometric mean (GM) geometric standard deviation (GSD) and range were calculated for the log normal distribution of all the measurements, including the static samples of the aerosol mass fractions, the concentrations of particle number and particle surface area (Table 1). A corresponding description, mean and range, of the blood concentration by sampling day is also presented (Table 2).

**Table 1:** Exposure concentration levels of inhalable dust and calculated personal exposure levels of inhalable, total and respirable dust, PM_10_, PM_2.5_, particle surface area (A-trak,) and particle number concentrations (P-trak) for all departments

Exposure concentration levels	*N*	AM	Median	SD	GM	GSD	Min	Max
Exposure measurements								
Inhalable dust (mg/m^3^)	72	0.30	0.14	0.52	0.15	3.1	0.005	3.3
Stationary measurements-calculated exposure levels								
Inhalable dust (mg/m^3^)	57	0.36	0.05	1.3	0.06	4.8	0.01	9.1
Total dust (mg/m^3^)	55	0.27	0.05	0.84	0.06	4.6	0.005	5.2
Respirable dust (mg/m^3^)	55	0.045	0.025	0.06	0.026	1.1	0.004	0.29
PM_10_ (mg/m^3^)	47	0.17	0.10	0.25	0.11	2.3	0.03	1.5
PM_2.5_ (mg/m^3^)	45	0.08	0.05	0.097	0.04	3.6	0.004	0.45
Total particle area (A-trak) (µm^2^/cm^3^)	55	110	38	200	19	18	0.001	1000
Particle number (P-trak)/cm^3^	55	41,000	19,000	60,000	12,000	9	22	270,000

*N* number of measurements, *AM* arithmetic mean, *SD* standard deviation, *GM* geometric mean, *GSD* geometric standard deviation

**Table 2:** Blood concentrations of biological markers, median (minimum–maximum) among 67 participants in different days

Biological marker	Afternoon	Pre-shift	Post-shift	Post-shift	Post-shift
	Day 0	Day 1	Day 1	Day 2	Day 3
Inflammation					
IL-1b (pg/ml)	2.3 (2.3–440)	3.4 (2.3–410)	2.3 (2.3–480)	2.3 (2.3–610)	2.3 (2.3–510)^a^
IL-6 (pg/ml)	4.8 (2.3–680)	7.7 (2.3–540)	5.7 (2.3–330)	4.1 (2.3–340)	4.2 (2.3–510)^a^
IL-8 (pg/ml)	5.1 (2.3–84)	6.1 (2.3–97)	4.3 (2.3–37)	3.9 (2.3–27)^b^	4.2 (2.3–31)^a^
IL-10 (pg/ml)	7.1 (2.3–1000)	8.2 (2.3–820)	5.5 (2.3–570)	6.3 (2.3–610)	6 (2.3–860)^a^
IFNγ (pg/ml)	2.3 (2.3–160)	2.3 (2.3–32)	2.3 (2.3–29)	2.3 (2.3–25)	2.3 (2.3–44)^a^
TNFα (pg/ml)	6.8 (2.3–160)	8 (3.5–130)	6.5 (2.3–160)	6.4 (2.3–200)	7.1 (2.3–170)
CRP (mg/l)	1.3 (0.13–27)	1.4 (0.16–48)	1.5 (0.16–42)	1.5 (0.15–21)	1.4 (0.18–28)
SAA (mg/l)	1.7 (0.13–65)	2.3 (0.39–73)	1.8 (0.016–64)	2 (0.001–58)	1.6 (0.001–63)
Fibrinogen (g/l)	2.7 (1.7–4.4)	2.7 (1.7–4.4)	2.7 (1.8–4.5)	2.7 (1.8–4.4)	2.7 (1.8–4.3)
Coagulation					
Faktor VIII (kIU/l)	0.98 (0.45–2.1)	1 (0.58–2)	1 (0.48–1.9)	0.99 (0.52–1.8)	1.1 (0.58–2)
vWillebrand (kIU/l)	1.1 (0.52–3.1)	1.1 (0.58–2)	1 (0.48–2.4)	0.98 (0.53–1.9)	0.99 (0.56–2)^b^
PAI (µg/l)	15 (4–80)	25 (5–80)	15 (2.9–66)	14 (2.8–68)	14 (2.7–80)^a^
D-dimer (µg/ml)	0.17 (0.11–1.2)	0.16 (0.11–1.5)	0.11 (0.11–1.5)	0.11 (0.11–2.1)	0.15 (0.11–11)

Five workers with infectious symptoms were excluded

^a^Biological marker with at least one post-shift significantly decreased compared to day 0

^b^Biological marker with at least one post-shift significantly increased compared to day 0

A linear mixed model was applied to describe the relation of the different inflammatory markers and the exposure metrics. Our design and use of mixed model offered a possibility to consider within-and between worker variability for our repeated measurements of inflammatory markers. We used the mixed model to study the biomarker levels between different days compared to day 0, the reference category (Table 2), and the variation of biomarkers by exposure categories 1–4 (Tables 3, 4).

**Table 3:** Mixed model analysis of particle exposure (*n* = 67) and inflammatory markers (*n* = 335) for 67 participants (five workers with infectious symptoms were excluded)

Exposure measure	CRP (mg/l)			SAA (mg/l)			Fibrinogen (g/l)		
	Mean	SE	*p*	Mean	SE	*p*	Mean	SE	*p*
Inhalable dust (mg/m^3^ p)									
Exp 4									
0.34+	2.77	1.35	**0.021**	3.13	1.35	0.104	2.87	1.06	0.231
Exp 3									
0.15–0.33	0.99	1.29	0.502	1.45	1.29	0.539	2.58	1.05	0.590
Exp 2									
0.08–0.14	1.29	1.37	0.909	2.69	1.37	0.227	2.82	1.06	0.348
Exp 1									
<0.08	1.24	1.31		1.79	1.31		2.67	1.05	
Inhalable dust (mg/m^3^ s)									
Exp 4									
0.15+	3.15	1.40	**0.000**	4.50	1.40	**0.004**	3.04	1.06	**0.007**
Exp 3									
0.06–0.14	1.60	1.35	**0.011**	2.12	1.35	0.150	2.80	1.06	**0.047**
Exp 2									
0.03–0.05	1.52	1.29	**0.015**	1.82	1.29	0.307	2.80	1.05	0.049
Exp 1									
<0.03	0.62	1.34		1.25	1.34		2.45	1.05	
Total dust (mg/m^3^ s)									
Exp 4									
0.14+	2.94	1.35	**0.007**	3.85	1.34	**0.038**	2.97	1.05	0.059
Exp 3									
0.06–0.13	1.32	1.34	0.556	1.60	1.34	0.708	2.80	1.05	0.311
Exp 2									
0.03–0.05	0.95	1.30	0.725	1.41	1.29	0.431	2.52	1.05	0.462
Exp 1									
<0.03	1.07	1.30		1.82	1.29		2.63	1.05	
PM10 (mg/m^3^ s)									
Exp 4									
0.14+	3.48	1.31	**0.009**	4.38	1.33	**0.004**	3.10	1.05	**0.015**
Exp 3									
0.08–0.13	0.99	1.29	0.178	1.62	1.30	0.889	2.62	1.05	0.612
Exp 2									
0.04–0.07	0.65	1.33	**0.019**	1.36	1.34	0.543	2.47	1.05	0.176
Exp 1									
<0.04	1.51	1.28		1.70	1.29		2.69	1.04	

Adjusted mean and SE by exposure classes 1–4 representing quartiles of the different exposure measures (particles size fractions). Inhalable personal, inhalable, total and P10 aerosol fractions

Bold value indicates *p* < 0.05

*p* personal sampling, *s* stationary sampling

**Table 4:** Mixed model analysis of particle exposure (*n* = 67) and inflammatory markers (*n* = 335) for 67 participants (five workers with infectious symptoms were excluded)

Exposure measure	CRP (mg/l)			SAA (mg/l)			Fibrinogen (g/l)		
	Mean	SE	*p*	Mean	SE	*p*	Mean	SE	*p*
Respirable dust (mg/m^3^ s)									
Exp 4									
0.06+	2.22	1.49	0.079	3.04	1.48	0.165	3.02	1.07	**0.038**
Exp 3									
0.03–0.05	1.89	1.32	0.075	2.39	1.31	0.278	2.78	1.05	0.206
Exp 2									
0.02–0.02	1.06	1.34	0.888	1.67	1.33	0.964	2.70	1.05	0.448
Exp 1									
<0.02	1.01	1.35		1.65	1.34		2.58	1.05	
PM2.5 (mg/m^3^ s)									
Exp 4									
0.09+	2.04	1.38	0.051	3.10	1.36	**0.014**	2.86	1.06	0.063
Exp 3									
0.05–0.08	1.35	1.36	0.416	2.36	1.34	0.090	2.72	1.05	0.296
Exp 2									
0.03–0.04	1.21	1.40	0.640	1.99	1.37	0.264	2.75	1.06	0.247
Exp 1									
<0.03	1.02	1.28		1.35	1.27		2.56	1.04	
Particle surface area (µm^2^/cm^3^ s)									
Exp 4									
96+	1.82	1.38	0.297	2.50	1.38	0.339	2.86	1.06	**0.038**
Exp 3									
47–96	1.41	1.30	0.778	1.93	1.31	0.851	2.76	1.05	0.157
Exp 2									
15–46	0.78	1.39	0.210	1.53	1.39	0.669	2.68	1.06	0.374
Exp 1									
<15	1.27	1.31		1.80	1.31		2.53	1.05	
Particle nr (nr/10^4^cm^3^ s)									
Exp 4									
3.19+	1.88	1.34	0.150	2.13	1.35	0.644	2.87	1.05	**0.038**
Exp 3									
1.81–3.18	1.58	1.29	0.357	2.14	1.30	0.638	2.81	1.04	0.093
Exp 2									
0.43–1.80	0.72	1.35	0.202	1.43	1.36	0.529	2.50	1.05	0.766
Exp 1									
<0.43	1.15	1.32		1.81	1.33		2.54	1.05	

Adjusted mean and SE by exposure classes 1–4, representing quartiles of the different exposure measures (particles size fractions). Respirable, PM 2.5, particle surface area and particle number air concentrations

Bold value indicates *p* < 0.05

*p* personal sampling, *s* stationary sampling

Since the distribution of inflammatory markers was skewed, a transformation to natural logarithm was performed. The estimates (*β*) of the fixed effects from the model allowed us to identify factors affecting inflammatory markers. The mixed model applied has the following equation

$$Y = \ln \left( X \right) = \mu + \beta_{1} \left[ {\text{EXPOSURE}} \right] + \beta_{2} \left[ {\text{DAY}} \right] + \beta_{3} \left[ {\text{GENDER}} \right] + \beta_{4} \left[ {\text{BMI}} \right] + \beta_{5} \left[ {\text{SMOKING}} \right] + \beta_{6} \left[ {\text{AGE}} \right] + \varepsilon$$

*Y*: ln(*X*); *X* and *Y* are the inflammatory marker concentration and log-transformed concentration
*µ*: the overall average inflammatory marker concentration on log-scale
*β*_1_: the fixed effects of exposure measures (*i*….*j*) classified by quartiles with the lowest exposure as reference category (exposure class 1)
*β*_2_: the fixed effects of sampling time (afternoon days 0, 1, 2, 3) with the afternoon day 0 (unexposed) as reference. Pre-shift morning values were not included in the analysis
*β*_3_: the fixed effects of gender, male or female with males as the reference category
*β*_4_: the fixed effects of BMI, dichotomized by median, the highest level used as reference category
*β*_5_: the fixed effects of smoking habits, ever, never and ex smoker, never smoker as reference category
*β*_6_: the fixed effects of age dichotomized by median, the oldest group as the reference category
*ε*: residual

The result of the mixed model analysis is presented as adjusted mean and standard error (SE). For statistical significance we used *p* < 0.05.

The analyses were performed after exclusion of five subjects with symptoms of infection, leaving 67 subjects for analysis. The analyses were performed with the SPSS software 22.0.

## Results

### Aerosols

Personal sampling of inhalable dust from 72 participants showed an 8-h TWA exposure of 0.30 mg/m^3^ with variation between 0.005 and 3.3 mg/m^3^, Table 1. The highest exposure was found in the mixing department, 3.3 mg/m^3^ and the average 8–h TWA exposure in this department was 1.6 mg/m^3^.

The calculated mean 8-h TWA of inhalable dust based on stationary measurement, 0.36 mg/m^3^, did not differ substantially from the mean of the personal measurements, 0.30 mg/m^3^, but the variation was larger for the stationary measurements.

### Markers of inflammation and coagulation

A series of blood samples from 67 exposed participants were obtained after the exclusion of five subjects with symptoms of infection. All measurements of biological markers of inflammation and coagulation are presented in Table 2. Our mixed model analysis showed significant decrease for most markers (IL-1b, IL-6, IL-8, IL-10, IFNγ, vWF, and PAI-1) was observed from the afternoon on day 0 and to one or several post-shift days in the workweek. However, there was one exception as factor VIII increased (Data not shown).

Some inflammatory markers showed several positive relations with exposure metrics, Tables 3 and 4. Increasing exposures, regarding the mass-based exposure measures, were related to significant increases in CRP, SAA and fibrinogen. Some of the highest quartiles of exposure of total dust showed a positive significant relation with several interleukins, e.g., IL-1b, IL-6, and IL-10, data not shown. Furthermore, a positive relation was also found between exposure to inhalable dust and IL-8. No significant relationships were found between the highest quartiles of exposures and IFNγ, TNFα, D-dimer, vWF, or PAI-1.

## Discussion

The main result of this study was the positive relation between several exposure metrics of the highest quartiles of exposure and some traditional markers of inflammation; CRP, SAA and fibrinogen. Correlations were found for the exposure metrics PM_10_, total and inhalable dust and these inflammatory markers. The correlations were generally stronger for larger particles, but some relations were also found for small particles (20 nm–1 μm). The consistent correlations between the exposure matrices (PM_10_, total and inhalable dust) and the well-established inflammatory markers (CRP, SAA and fibrinogen) supports a relationship between particle exposure and inflammatory markers. We did not observe any increase on a group level for most of the markers of inflammation or coagulation for the workers in this industry during a working week after a non-exposure period of 5 days.

To our knowledge, no data have been presented regarding the relationship between occupational exposure to particles and inflammatory markers in the pulp and paper industry, despite the observed risk of cardiovascular disease within this industry. The strength of this study is the measurement of airborne particles comprising a wide range of sizes from many different departments in the industry. However, the chemical composition has not been analyzed and the composition may differ somewhat in different departments, i.e., wood and paper dust, terpenes and bleachery chlorine-based chemicals, calcium hydroxide, sulfur dioxide, hydrogen sulfide, and various reduced sulfur compounds.

The significant decrease in most biological markers (IL-1b, IL-6, IL-8, IL-10, IFNγ, vWF, and PAI-1) is not well understood. A diurnal variation may play some role as the blood samples were taken 1–2 h earlier during the workweek (Rudnicka et al. 2007; Vgontzas et al. 2005).

CRP is the most sensitive acute-phase reactant and has widespread clinical use because serum CRP reflects the presence and intensity of inflammation (Li et al. 2012). In the review by Li et al. (2012), significant positive associations between ambient particle exposure and CRP were found in all four cross-sectional studies of children younger than 18 years. In contrast to the studies of children, weaker associations were found in cross-sectional studies conducted on adults. Three occupational studies were identified. Among 37 steel production plant workers, CRP levels were significantly positively correlated to the air concentrations of PM_1_ (median 3.6, range 1.7–30.5 μg/m^3^) and coarse PM (median 162, range 71–1190 μg/m^3^) (Bonzini et al. 2010). In a study of welders, a significant increase in CRP was observed 1 day after a mean exposure of PM_2.5_ at 1.66 mg/m^3^ (median 1.69, 25th–75th percentile 0.89–2.44) (Kim et al. 2005). In our previous study of workers exposed to particles, generated during processes of welding, cutting, grinding or foundry and concrete work, a relationship was found between particle exposure and CRP the second day of work after a summer vacation. The mean total dust exposure was 0.93 mg/m^3^ (range 0.034–6.3 mg/m^3^), (Ohlson et al. 2010). CRP also increased among volunteers exposed for 3–5 h to swine dust (Larsson et al. 1994). Thus, several occupational studies observed a relation between particle exposures and increased levels of CRP.

In the current study, several exposure metrics were correlated with plasma fibrinogen concentrations. The concentration of fibrinogen was not related to any of the measured aerosol fractions in our previous study (Ohlson et al. 2010). Other studies have shown increases after exposure to swine dust (Sjögren et al. 1999), concentrated ambient particles (Ghio et al. 2003), and urban air pollutants in Taipei (Chuang et al. 2007). However, there are some studies observing a significant decrease in fibrinogen in relation to air pollutant exposure. A significant decrease in fibrinogen was observed 6-h post-exposure in non-smoking welders (Kim et al. 2005). A significant decrease in both fibrinogen and thrombocytes was seen in Belfast and Edinburgh. This was discussed as a possible early consumption coagulopathy (Seaton et al. 1999).

The possible relation between air pollutants and SAA is less frequently studied (Ruckerl et al. 2011). However, one study of patients with coronary heart disease found a small positive relation between ambient particulate air pollution and SAA (Ruckerl et al. 2006).

In our previous study of industrial workers exposed to particles, generated during processes of welding, cutting, grinding or foundry and concrete work, a relationship was found between particle exposure and plasma levels of IL-6 the first day of work after a summer vacation (Ohlson et al. 2010). The workers in the present study were exposed to lower levels of particles in the pulp and paper industry (mean inhalable dust 0.30 mg/m^3^) compared to the workers of the previous study (mean total dust 0.93 mg/m^3^). Another difference between these two studies is the length of non-exposure period prior to the study. In the present study, the period was generally 5 days and in the previous study it was 4–5 weeks. A long exposure-free interval and a relative high exposure may generate an IL-6 response which is not observed when the exposure-free interval is short and the airborne exposure is relatively low. These two aspects may explain a weak response in the current study which was only significant in the highest quartile of total dust exposure.

Different responses were observed when previously unexposed volunteers and swine farmers were exposed for 3 h in swine confinement building weighing pigs. IL-6 increased fourfold among the volunteers but only slightly among the farmers with previous recent exposure to this environment (Palmberg et al. 2002). The inflammatory response investigated by bronchiolar alveolar lavage (BAL) was stronger among volunteers exposed once to zinc oxide fume 5 mg/m^3^ compared with volunteers exposed on three successive days to the same fume. The concentration of IL-6 in BAL was three times higher after the single exposure (Fine et al. 2000). These experiments indicate an adaptation with a milder inflammatory response after recent repeated exposures.

The highest afternoon concentrations of IL-8 and IL-10 were found on day 0, and the levels were found to be lower in all exposed afternoons. The pattern of this change is somewhat surprising as IL-8 is a pro-inflammatory chemokine which increased in bronchial wash after human exposure to diesel exhaust (Stenfors et al. 2004). IL-10 is a pleiotropic, immune regulatory cytokine that is important in protecting the host from infection-associated diseases, autoimmunity and allergy (Ng et al. 2013). However, we observed a positive significant relation between the highest quartile of inhalable dust exposure and IL-8 and also a positive significant relation between the highest quartile of total dust exposure and IL-10.

Some studies of pulp and paper mill workers have observed an increased risk of cardiovascular mortality (Hammar et al. 1992; Jäppinen and Tola 1990; Langseth and Kjaerheim 2006; Milham and Demers 1984; Persson et al. 2007; Torén et al. 1996). However, to our knowledge, no studies have presented adequate measurements of airborne pollutants and in order to perform dose-response or dose-effect calculations.

An increase in inhalable dust concentration with 0.84 mg/m^3^ (the mean of lowest quartile 0.05 mg/m^3^ versus the mean of the highest quartile 0.89 mg/m^3^) was associated with an increase in serum CRP concentration with 1.5, from 1.24 to 2.77 mg/l, Table 3. An increase in CRP from less than 0.9 mg/l to above 2.4 mg/l has been associated with a doubled risk of developing coronary heart disease (Danesh et al. 2000). However, the present study compares means of CRP concentrations and the previous study by Danesh compares the highest versus the lowest tertile represented by two cut offs. Consequently, the tentative extrapolated risk of coronary heart disease in the present study is lower than twofold.

The overall evidence from time-series analyses regarding urban air pollutants conducted worldwide has confirmed the existence of a small but consistent association between increased cardiovascular mortality of 0.4 % and short-term 10 µg/m^3^ elevations of PM_10_. Long-term exposures over years increase the risk for cardiovascular mortality to an even greater extent than exposures over a few days (Brook et al. 2010). An average exposure of 0.89 mg/m^3^ will theoretically increase the short-term cardiovascular mortality by 36 % (890/10 × 0.4 %).

This study shows consistent and significant increases in some inflammatory markers (CRP and SAA) when dust exposure (inhalable stationary measured dust, total dust and PM_10_) was 0.14 mg/m^3^ or higher. It could be argued that measurements from personal sampling of inhalable dust are better measures of individual exposures than the calculated exposure based on time spent in different work areas. However, the only significant positive relationship was between the inhalable dust levels of 0.34 mg/m^3^ or above and CRP, Table 3.

## Conclusion

We have determined air exposure to particle mass, particle number and particle surface area air concentrations and inflammatory markers. The mean 8-h TWA of inhalable dust exposure was 0.30 mg/m^3^, and the range was 0.005–3.3 mg/m^3^. Several of the exposure metrics were associated with dose-dependent increases in some inflammatory markers in the blood (CRP, SAA, and fibrinogen). These dose-effect relations may indicate an increased risk of cardiovascular disease. We did not observe any increase on a group level for most of the markers of inflammation or coagulation for the workers in this industry during a working week after a non-exposure period of 5 days. Our study group is relatively small, and the findings need to be confirmed by investigating larger number of exposed workers in the pulp and paper industry.
